# Both Barriers and Trait Complementarity Govern Pollination Network Structure

**DOI:** 10.1371/journal.pbio.0050054

**Published:** 2007-01-23

**Authors:** Richard Robinson

A field of spring wildflowers, abuzz with busy insects seeking nectar and spreading pollen, may look like a perfect model of random interaction. But ecologists have discovered order within this anarchy. For instance, as the number of species grows, the number of interactions does too, while the connectivity (the fraction of possible interactions that actually occur) and the nestedness (the relative importance of generalist species as mutualistic partners of specialist species) shrinks. Study of such networks of species is still in its youth, and the rules that generate these patterns of interaction are still being worked out. In a new study, Luis Santamaría and Miguel Rodríguez-Gironés propose that two key mechanisms—trait complementarity and barriers to exploitation—go a long way in explaining the structure of actual networks of plants and their many pollinators.

The two mechanisms each arise from fundamental aspects of the interaction between species. An insect will be unable to reach nectar in floral tubes longer than its proboscis: the tube length sets up a barrier to some species, but not to others. Each plant species also has a given flowering period: the specific activity period of each insect species will complement the flowering of some plant species more than others. Other barriers and other complementary traits have been described for a variety of plant–pollinator pairs.

To explore the significance of these mechanisms, the authors modeled plant–pollinator interaction networks using a few simple rules, and compared their results to data from real networks in real plant communities. Barrier traits were described by a single measurement (akin to length of floral tube or proboscis). In the model, an insect interacted with a plant if its trait measurement exceeded the plant’s trait measurement. Complementary trait values (akin to plant and pollinator phenologies) had to roughly match in order for the two species to interact. The models incorporated from one to four barrier or complementary traits, or a combination of two of each. They also tested two variations of a “neutral” interaction model, in which species interact randomly, based simply on their relative abundance.

Different models did better at mimicking different aspects of real networks, but the two that performed best overall were the combination model and one of the neutral models. The authors argue that the neutral model, despite its appealing simplicity, can be discounted because it requires key assumptions regarding species abundances and random interaction that conflict with empirical observations of real communities.

In contrast, the model combining barriers and complementary traits matches well with observed plant–pollinator interactions. Barriers alone would mean that pollinators with the longest proboscis would be supreme generalists, able to feed on any flower, causing perfect network nestedness; while complementarity alone would mean that specialist pollinators do not interact primarily with generalist plants, causing unrealistically low network nestedness. Instead, the authors suggest, a combination of barriers and complementary traits accounts for the pattern of specialists and generalists seen in real pollination networks.

**Figure:**
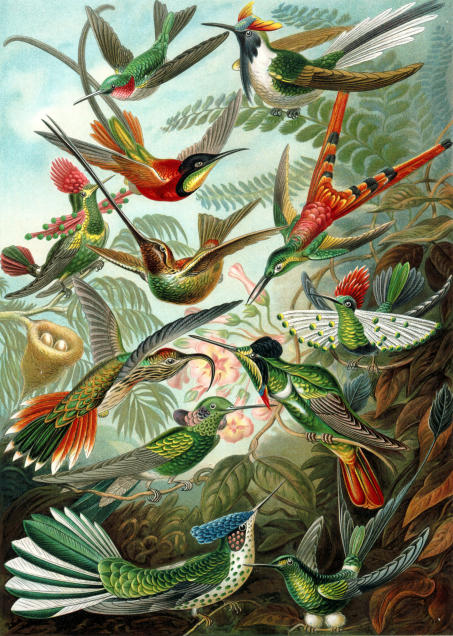
Researchers are just beginning to understand the mechanisms governing the complex network interactions between plants and pollinators, such as hummingbirds, shown in this illustration from Ernst Haeckel's *Kunstformen der Natur* (1904).

The superiority of the combination model also has implications for understanding floral evolution. A common principle has been that plants coevolve with their most-efficient pollinator to strengthen the complementarity of their matching adaptations. Barriers, however, while reducing exploitation by inefficient pollinators, may also interfere with pollination by efficient ones. Nonetheless, the results of the present study indicate that barriers are likely to play an important role in pollinator networks, suggesting that coevolution with the most-efficient pollinator is not the sole factor governing floral evolution.

